# Modern Isms and Ologies, (Poetical)

**Published:** 1874-11

**Authors:** 


### *MODERN ISMS AND OLOGIES.*

The following ode gives a witty expose of the condition of the different schools of philosophy. The refrain is, of course, to be repeated by those who like it, after each stanza:

A DIDACTIC ODE.—*To be sung to the tune of "Guy Faux."*

Ours is a wise and earnest age, an age of thought and science, Sir;
To error, ignorance and bliss we fairly bid defiance, sir.
"Professors" everywhere abound, both in and out of colleges,
And all agog to cram our nobs with "isms" and "ologies."

Bow, wow, wow,
Tol de riddle, tol de riddle,
Bow, wow, wow.

Philosophy, as you're aware, material and mental, Sir,
At one extreme is "Positive," at t'other "Transcendental," Sir;
And each of us who in these days would speculate "en regle,"
If he can't run the rig with Comte, must take the tip from Hegel.

The fundamental problem which, debated now for ages, Sir,
Is still attacked and still unsolved by all our modern sages, Sir,
Is, if an effort I may make a simple form to throw it in,
Just what we know, and why we know, and what's the way we know it in.

We can't assume (so Comte affirms) a first or final cause, Sir.
Phenomena are all we know, their order and their laws, Sir;
While Hegel's modest formula a single line to sum in,
Is "nothing is and nothing's not, but everything's becomin."

"Development" is all the go, of course, with Herbert Spencer,
Who cares a little more than Comte about the "why" and "whence," Sir.
Appearances, he seems to think, do not exhaust totality,
But indicate that underneath there's some "Unknown Reality."

And Darwin, too, who leads the throng "in vulgum voges spargere,"
Maintains Humanity is naught except a big menagerie,
The progeny of tailless apes, sharp-eared but puggy-nosed, Sir,
Who nightly climbed their "family trees," and on the top reposed, Sir.

There's Carlyle, on the other hand, whose first and last concern it is
To preach up the "immensities" and muse on the "eternities;"
But if one credits what one hears, the gist of all his brag is, Sir,
That "Erbwurst," rightly understood, is transcendental "Haggis," Sir.

Imaginative sparks, you know, electric currents kindle, Sir,
On Alpine heights, or at Belfast, within the brain of Tyndall, Sir;
His late address, some people hold, is flowery, vague, and vapory,
And represents the "classic nude" when stripped of all its "Draper"-y.

Professor Huxley essayed to bridge across the chasm, Sir,
'Twixt matter dead and matter quick by means of "protoplasm," Sir,
And to his doctrine now subjoins the further "grand attraction"
That "conciousness" in man and brute is simply "reflex action."

Then Stanley Jevons *will* contend in words stout and emphatical
The proper mode to treat all things is purely mathematical;
Since we as individual men, communities, and nations, Sir,
Are clearly angles, lines, and squares, cubes, circles, and equations, Sir.

George Henry Lewes, I'm informed, had "gone off quite hysterical"
About that feeble, foolish thing, the "theory Metempirical;"
And only found relief, 'tis said, from nervous throes and spasms, Sir,
By banging straight at Huxley's head a brace of brand-new "plasms," Sir.

Such are the philosophic views I've ventured now to versify,
And, if I may invent the term, in some degree to "tersify."
Among them all, I'm bold to say, fair room for choice you'll find, Sir,
And if you don't, why then you won't, and I for one sha'n't mind, Sir.

