# Amende

**Published:** 1874-12

**Authors:** 


AMENDE.—By oversight, in our last issue we omitted to credit the Ode on page 481 to the *Philadelphia Medical Times.* We now hasten to do this act of simple justice, and to apologize for any other omissions of like character. We are quite willing that others should credit our own bantlings to an imaginary "Ex," or even appropriate them as original matter, but we are very unwilling to appear in such category ourselves.

